# Modelling the Pleistocene colonisation of Eastern Mediterranean islandscapes

**DOI:** 10.1371/journal.pone.0258370

**Published:** 2021-10-27

**Authors:** Theodora Moutsiou, Christian Reepmeyer, Vasiliki Kassianidou, Zomenia Zomeni, Athos Agapiou

**Affiliations:** 1 Archaeological Research Unit, University of Cyprus, Nicosia, Cyprus; 2 ARC Centre of Excellence for Australian Biodiversity and Heritage, College of Arts, Society and Education, James Cook University, Cairns, Australia; 3 Department of Archaeology, Max Planck Institute for the Science of Human History, Jena, Germany; 4 Science and Technology in Archaeology and Culture Research Center, The Cyprus Institute, Nicosia, Cyprus; 5 Geological Survey Department, Lefkosia, Cyprus; 6 Department of Civil Engineering and Geomatics, Faculty of Engineering and Technology, Cyprus University of Technology, Limassol, Cyprus; 7 Eratosthenes Centre of Excellence, Limassol, Cyprus; University at Buffalo - The State University of New York, UNITED STATES

## Abstract

Predictive models have become an integral part of archaeological research, particularly in the discovery of new archaeological sites. In this paper, we apply predictive modeling to map high potential Pleistocene archaeological locales on the island of Cyprus in the Eastern Mediterranean. The model delineates landscape characteristics that denote areas with high potential to unearth Pleistocene archaeology while at the same time highlighting localities that should be excluded. The predictive model was employed in surface surveys to systematically access high probability locales on Cyprus. A number of newly identified localities suggests that the true density of mobile hunter-gatherer sites on Cyprus is seriously underestimated in current narratives. By adding new data to this modest corpus of early insular sites, we are able to contribute to debates regarding island colonisation and the role of coastal environments in human dispersals to new territories.

## Introduction

Recent advances in the study of island colonisation, which showed the scale of hunter-gatherer interaction with maritime resources and island landscapes [1–6], require us to revisit the question of a potential Pleistocene human presence on Cyprus. Unequivocal evidence of material culture of this period is currently largely absent from Cyprus, but recent research has shown that an earlier colonisation of the island is viable [7]. Moreover, the current absence of evidence might relate to missing research focus on earlier time periods rather than representing an archaeological reality. This paper presents a methodological approach that combines traditional archaeological practices and technological applications from the field of geoinformatics as a tool for the investigation of a potential Pleistocene colonisation of the island of Cyprus. Although our emphasis is the Late Pleistocene (last 125 ka), we envisage that such an approach will be a useful means in the identification of archaeological sites of other periods too, particularly in areas, where no archaeological sites have been found so far.

Here we report on a study designed to systematically investigate high potential localities for Late Pleistocene human presence either for purposes of habitation or exploitation of subsistence resources (e.g. food, raw materials). The geospatial modelling of target areas is presented here functioning as the starting point for a large-scale research-based field survey aimed at collecting empirical archaeological data that will feedback into the model. A simple set of assumptions regarding Palaeolithic decision-making underpin this study; fundamentally, we assume that although limited concrete evidence for Pleistocene archaeology exists on the island, multiple Pleistocene mobile hunter-gatherer groups could have entered Cyprus, either on short-term forays or longer stays. In addition to hunting medium-sized game and using good quality chert for stone tool making, we assume that they were familiar with coastal and marine resources, also present on Cyprus. This assumption stems from recent studies that document a generalised subsistence system, including coastal foods, for early humans [8, 9]. Thirdly, we assume that Pleistocene hunter-gatherers could have used all regions of the island, including mountainous areas. We base this assumption on the fact that Terminal Pleistocene and Early Holocene sites on Cyprus with hunter-gatherer-forager elements are found located not only coastally but also inland and upland, such as Vretsia *Roudias* [10] and Krittou Marottou *Ais Giorkis* [11].

### Predicting locales of past human activity

Predictive modeling is a method used to identify archaeological site locations on the basis of observed patterns and assumptions about human behaviour [12–15]. It was initially developed in the mid-1970s and mainly applied within the context of governmental land management and Cultural Heritage management [12, 16, 17]. Early predictive models have focused on the application of multivariate statistical techniques to discuss site and settlement patterns by extrapolating from sample to population [12]. More recently, digital predictive methodologies combining Geographical Information Systems (GIS) and geostatistical methods have been used to estimate the probability of encountering archaeological sites outside of areas where they have already been found in the past. Spatial analysis and predictive modeling using GIS have been subjected to fierce debate regarding the suitability of such approaches in archaeological research. Predictive models have been heavily criticised as leading to environmental determinism in archaeology [18–21] and often considered as unable to predict the location of all archaeological sites [22, 23]. Despite its shortcomings, predictive modeling has proved a valuable tool for the rescue of archaeological remains and data that would have otherwise been lost due to modern development. Beyond Cultural Heritage management applications, predictive modeling has already been successfully exploited in spatial planning of archaeological research [24, 25]. Predictive models have resulted in particularly useful insights especially in areas where there is an extreme paucity of data that could be used as a baseline for expanding archaeological work or in cases where extensive areas needed to be covered with traditional surveys [26–28]. In such instances, predictive models can assist in the determination of which regions are more likely to produce archaeological localities and which regions are less likely to do so.

In our study, the focus is predominantly on low-density lithic scatters—the main material remains of prehistoric hunter-gatherer activity. Such archaeological signatures are largely ephemeral with low visibility and, hence, particularly difficult to identify in the landscape (see [7] for a detailed discussion). It is of paramount importance, then, to pay a closer look at the insular landscape in order to understand how Pleistocene hunter-gatherers would have inhabited and used their environment. Here we combine environmental and socio-cultural factors, focusing on features of the landscape that would have had socio-cultural significance for early hunter-gatherers, such as raw material sources.

## Regional setting

Cyprus is the third largest island in the Mediterranean extending over 9,251 km^2^ and at distances around 30–70 km (depending on marine transgressions) from the nearest coastline. It is also a resource-rich island, encompassing good quality lithic sources and an ecosystem that could sustain hunter-fisher-gatherer groups [7]. Despite all this, however, Cyprus has remained at the periphery of early island colonisation discussions mainly due to an ongoing assumption of its marginality due to open water gaps functioning as barriers to pre-Holocene maritime crossings (see discussion in [29]). Global interest in early seafaring and maritime colonisation in recent years [1, 2, 30] has led to significant advances in the thematic and there is tentative evidence that *Homo sapiens* as well as pre-sapiens species were capable of maritime crossings and successfully colonised insular environments in various parts of the world (see [27, 31, 32] for some eastern Mediterranean examples).

### Archaeological background

Akrotiri *Aetokremnos* [33, 34] in southern Cyprus is currently the oldest securely dated site on the island with dates that place its occupation at the Terminal Pleistocene (ca. 12 ka). The coastal sites of Nissi Beach and Akamas *Aspros* [35], and Vretsia *Roudias* [10] and Ayia Varvara *Aspokremmos* [36, 37] further inland, add to the number of likely Early Holocene sites with hunter-gatherer/forager attributes. The first permanent settlements, for example Ayios Tychonas *Klimonas* [38] also appear during the Early Holocene (ca. 10 ka) followed by a Neolithic settlement expansion well into the Middle Holocene (ca. 8 ka).

**Fig 1:**
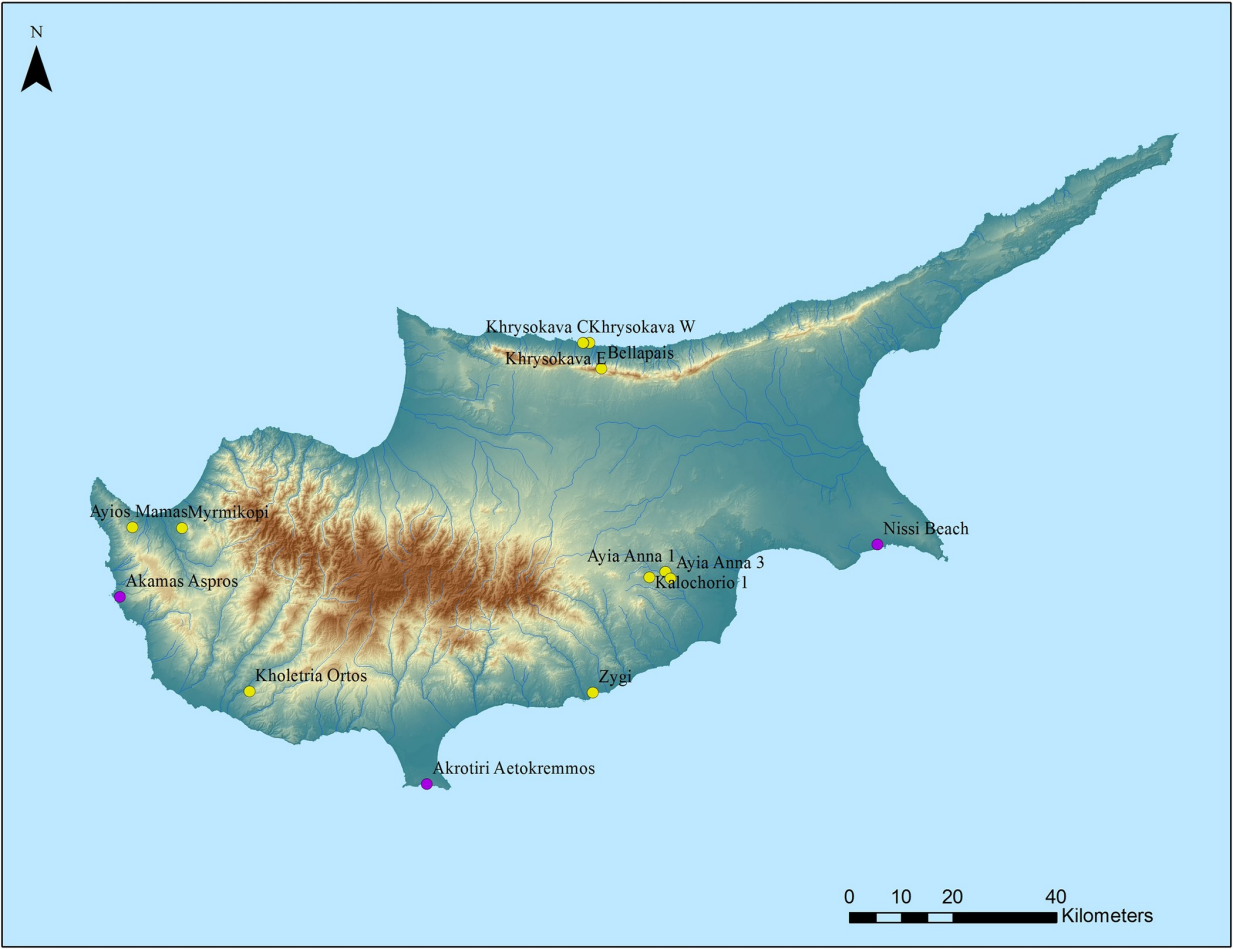
Terminal Pleistocene sites and legacy Palaeolithic surface lithic assemblages from Cyprus. Map depicting known Terminal Pleistocene archaeological sites and localities where legacy Palaeolithic lithic assemblages (with typo-technological characteristics ranging from late Middle to Late Pleistocene) have been collected across Cyprus used in the development of the predictive model. Compiled in ArcGIS with Digital Elevation Model (DEM) data from the Land and Surveys Department of the Republic of Cyprus. Original copyright with the authors.

Beyond these excavations, only small collections of surface lithic finds of a tentative Palaeolithic character [7] have been documented from various parts of the island during the 1960s-1980s (Fig 1). Stockton ascribes an Upper Palaeolithic character to a lithic assemblage, including cores, flakes and formal tools (86 lithics), which he collected from four different localities at the Kyrenia range, on the northern coast of Cyprus, near the area of Khrysokava during a short visit to the island in 1967. In 1968, during a brief visit to the island, Vita-Finzi [39] collected a small assemblage of lithic artefacts from a fossil beach near the mouth of the Maroni River at Zygi (Larnaka District), southeast Cyprus. The artefacts were assigned to the Middle Palaeolithic, based on their typo-technological characteristics and geological context. The archaeological reconnaissance of the Khrysochou Bay area project [40] and the Tremithos Valley Project [41] targeting the Khrysochou drainage near Polis (Paphos District), in northwest Cyprus, and the Tremithos Valley (Larnaka District), southern Cyprus, respectively, documented amongst other material culture, lithic collections with typo-technological characteristics of a potential Palaeolithic age. Finally, in 2016 Strasser et al. [42] ascribed a Lower Palaeolithic age to a biface, a surface find which was collected by S. Swiny in 1992 near the Aceramic Neolithic site of Kholetria *Ortos* (Pafos District), southwest Cyprus [43], after examining a three-dimensional printed replica of the artefact. The significance of these finds in demonstrating a Pleistocene human presence in Cyprus, either in the form of ephemeral visits or a more permanent habitation, is, nevertheless, limited. Surface finds are unreliable age markers, especially when dealing with extremely small numbers. Additional data are needed to add to this modest corpus of information and fast-track research into the Pleistocene past of the island.

## Materials and methods

A key problem in predictive modeling is the availability of representative archaeological data that can be used either as input, or as a test for a set of expectations [44]. Cyprus has a limited dataset related to Pleistocene archaeology. To counterbalance this paucity, we have utilised data from better-studied regions within the Eastern Mediterranean region. Essentially, we correlate the locations of known sites with landscape and topographic features and use this information to model locations of other unknown sites in an area. The list of sites used in the analysis is included in S1 Table. Although the actual number of archaeological sites in these areas might be underestimated, we consider these as representative site samples sufficient for our model. Limitations of this approach are discussed in the appropriate section below.

### Variables

Here we consider parameters on the Cypriot landscape that would have been attractive to early humans: freshwater sources, raw material sources and topographical features. Freshwater sources apart from being vital to human survival would have attracted fauna also necessary for human subsistence; sources of good quality raw materials are essential for the manufacture of a reliable tool-kit. Topographical features of relevance include slope, aspect and elevation. Mobile hunter-gatherers of the Late Pleistocene would have been familiar with coastal resources and marine foods [9] but not restricted to such environments even when on islands as often assumed. In order to account for this, we also consider distance from the (modern) coast and fresh water sources, such as rivers and springs. We considered all these variables initially independently from each other to determine whether any given variable is preferentially selected. Once it became clear that we do not have any distinguishable patterns, we used these variables together in developing our model to avoid biases in estimates of importance (see also [12]). The model was developed using various methods: Boolean geometry, proximity estimation and similarity analysis. We performed our GIS analyses based on two spatial input-data variables: local and regional.

#### Regional-based data analysis

We collected data from various published sources [45–48], including material from surveys and excavated horizons from mainland Greece, the Ionian and Aegean Seas, Turkey, Israel, Syria, Jordan and Lebanon (S1 Table). Data and maps from these reports were georeferenced and then digitised in a GIS environment for further analysis.

#### Local-based data analysis

On a local-based data analysis, which encompasses the full extent of the island of Cyprus, we concentrated on the localities of the documented legacy collections of a potential Palaeolithic character. Furthermore, taking into consideration that hunter-gatherers exploited Cyprus at the end of the Pleistocene, we opted to include known Epipalaeolithic sites dating to the Terminal Pleistocene in our analyses (Fig 1).

### Data

The geospatial analyses concerning the broader Eastern Mediterranean relied upon global Digital Elevation Models (DEM) from the Shuttle Radar Topography Mission (SRTM) Digital Elevation Database. For the geospatial analyses concerning Cyprus we make use of the DEM, acquired from the Department of Land and Surveys of Cyprus (DLS). In addition to the DEMs, other geographical data such as major rivers in the Eastern Mediterranean region and the drainage maps of Cyprus have been used.

### The model

The predictive model was created using a combination of layers for elevation, slope, aspect, distance from rivers/springs and the coast, as well as lithology and general geology on the local scale. In choosing these parameters, we consider the natural environment but also cognitive and behavioural aspects of Pleistocene hunter-gatherers. As such, our approach follows a theoretically informed data-driven modeling. We expect our analyses to result in a robust model for the investigation of Pleistocene localities in Cyprus but there remain some limitations. The most obvious one is inherent in any GIS approach, namely that in the application of geospatial tools, we are estimating past landscapes with features we can only quantify in the present (for a discussion, see the ‘complex topography concept’ [49, 50]). Moreover, remotely sensed data can only record the present surface and very shallow subsurface topography.

In coastal regions the onset and offset of 60+ ice ages, which characterise the Quaternary led to extreme sea-level changes (as much as 120 m sea-level drop during ice ages and as much as 6m sea-level rise during interglacial periods, both in relation to present sea-level), resulted in substantial impacts to near-shore landscape morphology. Phenomena such as tectonics, isostacy and changes in erosion patterns have all contributed to the Quaternary being a geologic period with extreme landscape changes, at least as it pertains to humans, their evolution and behaviour in relation to the environment [51, 52].

**Fig 2:**
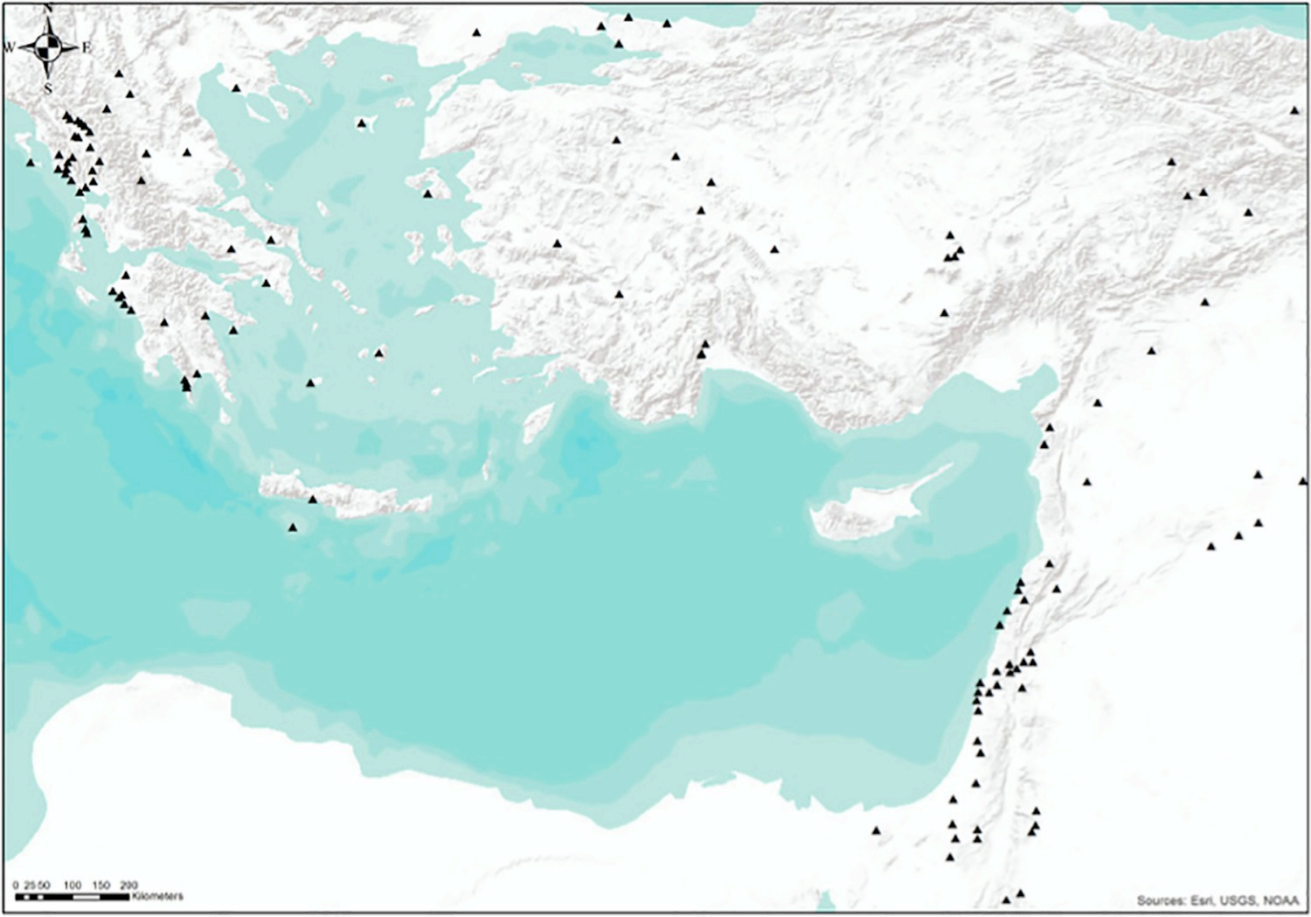
Eastern Mediterranean Pleistocene sites. Map showing Pleistocene archaeological sites in the circum-Cyprus Eastern Mediterranean region. A detailed list of the sites and references can be found in S1 Table. Original copyright with the authors.

In the first iteration of the model, our input-data were based on a regional scale (Fig 2) and, particularly, on topographic variables in order to determine whether any clear patterns regarding the location of Pleistocene sites within the broader Eastern Mediterranean emerge. For this, we analysed over 100 archaeological localities from the circum-Cyprus region, specifically looking at their elevation, slope, aspect and distance from rivers and the sea. The attributes of all the analysed Eastern Mediterranean sites fed back to the predictive model used for Cyprus.

In the application of the model to Cyprus, we tested the results of the regional model on a local scale and incorporated information from legacy Palaeolithic surface collections and known hunter-gatherer, Epipalaeolithic, sites on Cyprus. Fifteen sites were plotted as shown in Fig 1 and Table 1. Their environmental attributes where then extracted and analysed. At this stage, we also incorporated additional variables, specifically smaller fresh water sources (springs), lithology and geology. In detail, the distance from coastline, rivers, elevation, aspect and slope was examined, as well as lithology and geological attributes. Initially, each parameter was evaluated separately to investigate their importance for the creation of a predictive model. However, this did not provide any clear patterns, and therefore a multi-variable analysis was processed using Boolean algebra. In addition, an alternative approach was followed: a probability map, based on one-class classification analysis, using the Spectral Angle Mapper (SAM) classifier was performed. The results eventually provided areas where there is a higher probability to share similar attributes with the ones of the fifteen earliest known archaeological locales of Cyprus. At the last stage, we also tried to limit the areas of future investigations taking into consideration the Quaternary (terrestrial) geology of the island ignoring the Holocene and focusing on the Pleistocene, including chert-bearing formations that would have provided raw materials for tool manufacture (see maps below).

**Table 1:** Main topographic features of Terminal Pleistocene and legacy Palaeolithic surface lithic assemblages from Cyprus.

Sites	Elevation (m, a.s.l.)	Slope (degrees)	Distance from Coastline (m)	Distance from Rivers (m)
Ayia Anna 3, Tremithos Valley	100–200 m	>20 degrees	>5000 m	100–500 m
Kholetria Ortos, 1 handaxe	200–350 m	10–20 degrees	>5000 m	500–5000 m
Kalochorio 1, Tremithos Valley	200–350 m	10–20 degrees	>5000 m	500–5000 m
Ayia Anna 1, Tremithos Valley	100–200 m	10–20 degrees	>5000 m	500–5000 m
Helicopter pad at Tjiklos, Kyrenia range	200–350 m	>20 degrees	500–5000 m	100–500 m
Bellapais	>350 m	0–10 degrees	500–5000 m	500–5000 m
Khrysokhou River drainage, Ayios Mamas	100–200 m	0–10 degrees	500–5000 m	500–5000 m
Nissi Beach	0–100 m	0–10 degrees	500–5000 m	500–5000 m
Khrysokava, east of Kyrenia	0–100 m	0–10 degrees	100–500 m	500–5000 m
Khrysokava, east of Kyrenia	0–100 m	0–10 degrees	< 100 m	500–5000 m
Khrysokava, east of Kyrenia	0–100 m	0–10 degrees	< 100 m	500–5000 m
Akamas Aspros	0–100 m	10–20 degrees	500–5000 m	500–5000 m
Fossil beach near Maroni River mouth, Zygi	0–100 m	0–10 degrees	500–5000 m	500–5000 m
Khrysokhou River drainage, Myrmikopi	100–200 m	0–10 degrees	500–5000 m	500–5000 m
Akrotiri Aetokremmos	0–100 m	>20 degrees	< 100 m	>5000 m

Main topographic features of Terminal Pleistocene archaeological sites and Palaeolithic surface assemblages on Cyprus.

### Field methodology

Field methodology followed a classic reconnaissance survey approach with a small team of experts targeting high probability areas as delineated by the predictive model. Permits for this fieldwork were acquired in written form by the Department of Antiquities of the Republic of Cyprus. The primary objective of the field surveys was to systematically search specific regions for remains of human activity and to collect surface archaeological samples to the validity of the predictive model and to demonstrate function and date of human activity. Between September and October 2020, the research team conducted systematic by-foot surface surveys, focusing on the areas/zones delineated by the model as ‘hotspots’ and especially targeting specific topographic features (see ‘Local’ Results section) in these zones, such as rockshelters, riverbanks and sediments of a known Pleistocene age, as more likely to preserve in situ material. All investigated areas/tracks were documented via tracklogs with GPS points and detailed soil and landscape descriptions (e.g. average surface visibility, present land use, and vegetation) recorded for localities that preserved cultural remains. Surface sampling was conducted in order to determine the function and age of the artefacts and the use of different parts of the area in different periods. A spatially well-defined concentration of lithic cultural materials (stone tools) was designated a findspot (lithic scatter). In areas of high interest, sediment depth was investigated through probing and augering in order to record points for possible future test excavations. At the end of the research, all the new data that have been georeferenced were collated in density/distribution maps.

## Results

### Regional

**Fig 3:**
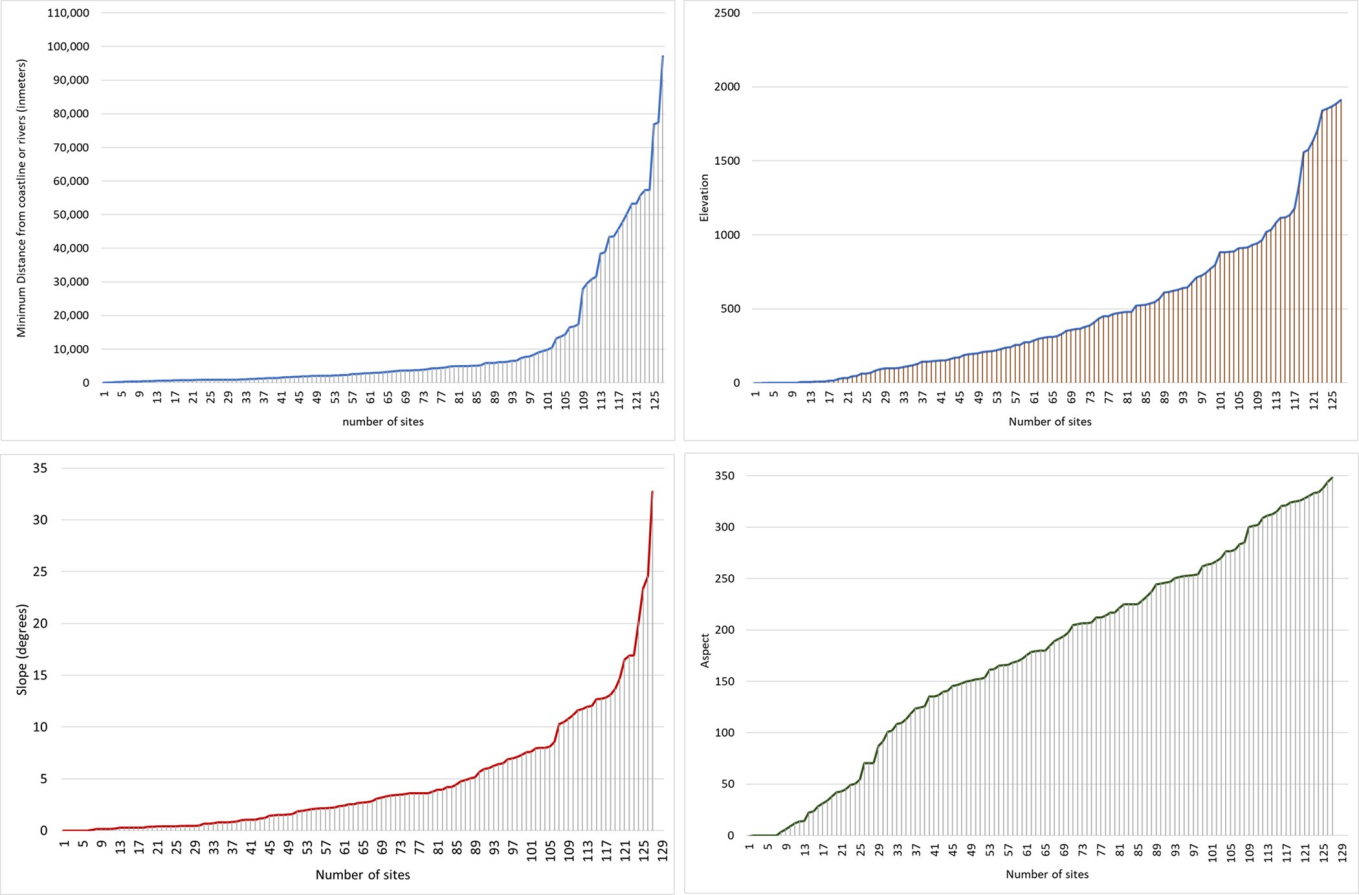
Main topographic patterns of Eastern Mediterranean Pleistocene archaeological sites. Graphs (a = distance from coast/rivers, b = elevation, c = slope, d = aspect) summarising the main topographic patterns of known Pleistocene archaeological sites across the Eastern Mediterranean.

The main aim of our model is to delineate a zone of high probability to unearth Pleistocene archaeological remains on the island of Cyprus that is as precise as possible. We started by modeling the main characteristics of known Pleistocene localities within the Eastern Mediterranean, expecting to define potential topographical patterns that may be used in delineating a probability zones on Cyprus. Fig 3A–3D summarises the main topographic patterns of the region. Slope, aspect and elevation do not appear to be significant factors in the location of Pleistocene sites in the Eastern Mediterranean. Aspect is particularly non-informative and elevation similarly so. Two thirds of the sites are at an elevation that reaches or exceeds the highest mountain ranges of Cyprus. Slope is somewhat more informative as it suggests a preference for locations with a slope component <20°, with the majority of the sites found at <15°. Regarding distances from water, the majority of the sites appear to be within 10 km (maximum distance) from either a river or the coastline. Sites are predominantly located closer to a river source than the coast. Sites found at a great distance from rivers are probably supplied with fresh water by smaller water sources not included in the available dataset. Overall, the regional scale analyses highlight the significance of freshwater proximity and the secondary nature of topographic features, especially aspect, in site location preferences.

### Local

**Fig 4:**
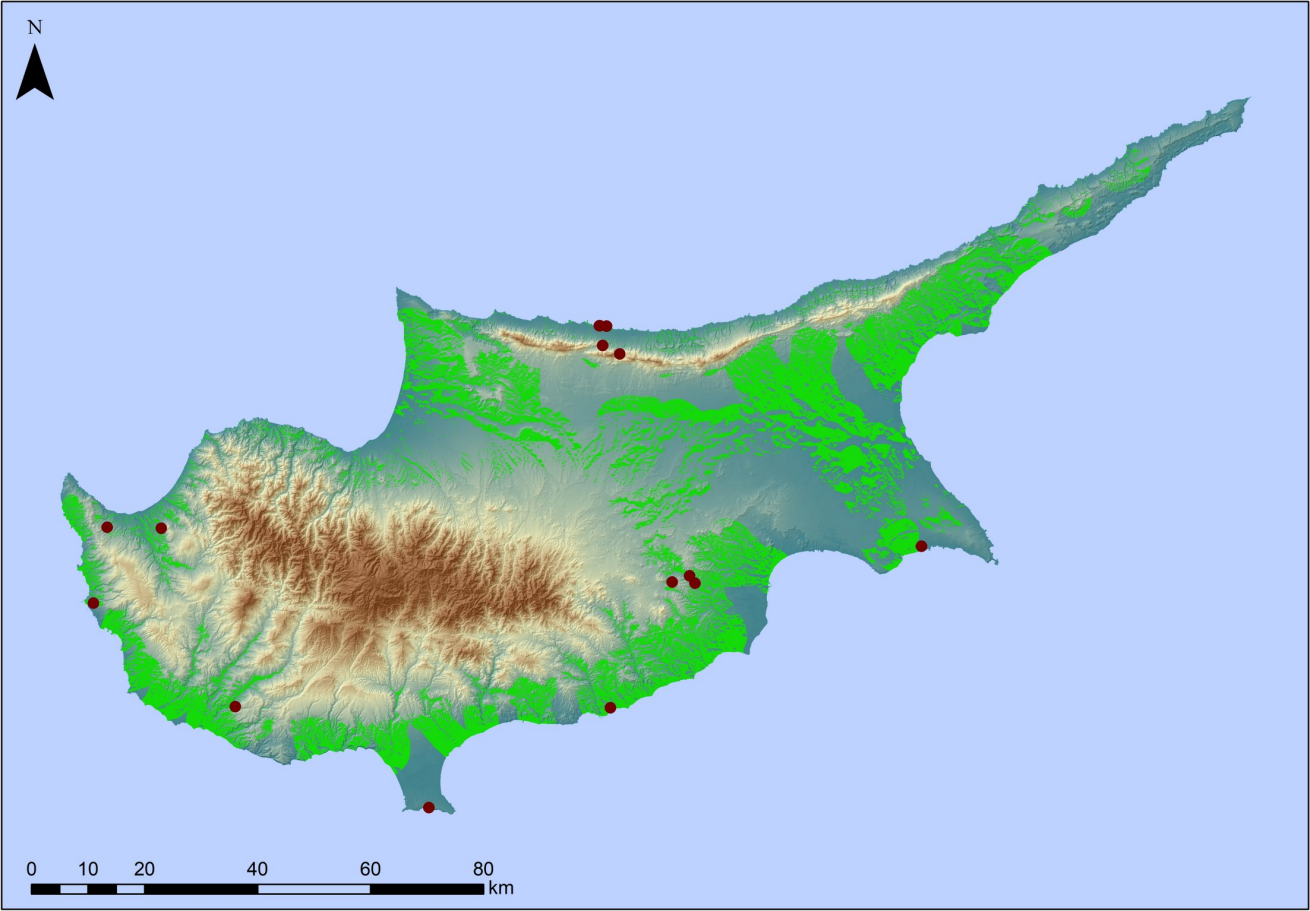
Prediction map of high probability areas for Pleistocene archaeological sites on Cyprus. Prediction map delineating high probability areas for the location of potential Pleistocene archaeological sites on Cyprus based on attractive topographic attributes. The dark red dots represent known early archaeological sites on Cyprus (see Fig 1 for site names). Note how all but one archaeological sites fall within the high probability zone (green area) as defined by the predictive model. Original copyright with the authors.

**Fig 5:**
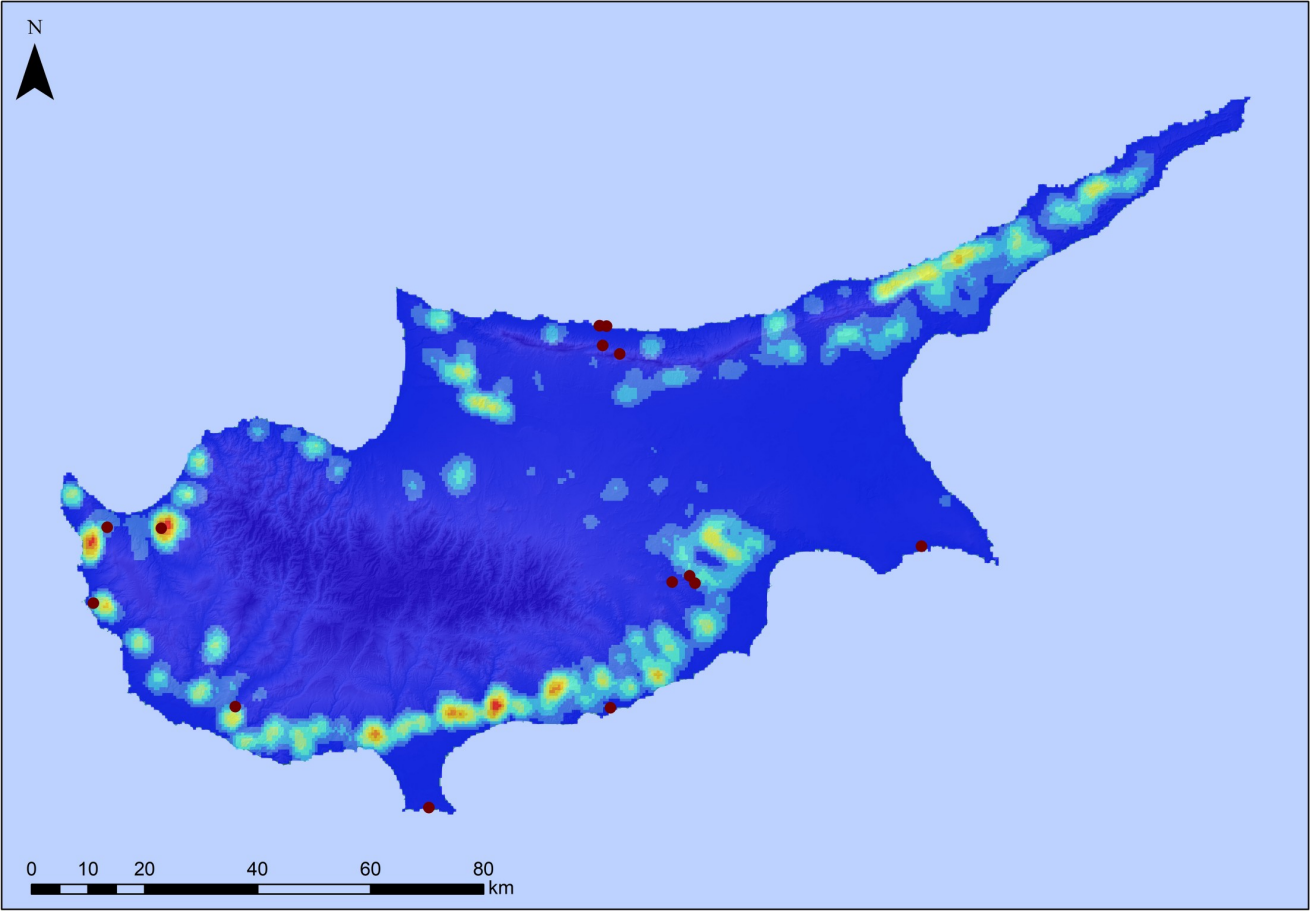
Probability map based on one-class classification analysis. Probability map based on one-class classification analysis, using the Spectral Angle Mapper (SAM) classifier. The map illustrates areas where there is a higher probability to share similar attributes with the ones of the fifteen earliest known archaeological sites of Cyprus. The dark red dots represent known early archaeological sites on Cyprus (see Fig 1 for site names). Original copyright with the authors.

**Fig 6:**
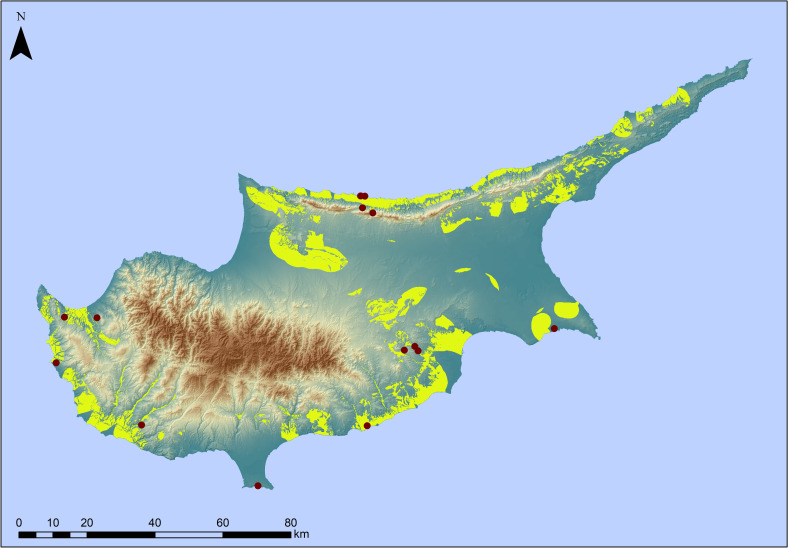
Prediction analysis final output. The map illustrates the final output of the predictive analyses, taking into account the combination of slope, elevation, aspect, distance from coast and rivers results as well as Pleistocene terrestrial geology and raw material availability. The map delineates the smallest possible area of high potential to preserve Pleistocene archaeology based on the currently available data (green zone). The dark red dots indicate the location of known early archaeological sites on Cyprus (see Fig 1 for site names). Original copyright with the authors.

In our next step we focused on Cyprus itself exploring patterns of Terminal Pleistocene archaeological sites and surface assemblages with typo-technological characteristics of a probable Palaeolithic age known from across the island (**Table 2**). Here too, aspect was found not to be informative. With regards to elevation most of the sites are located <350 m with 46.6% of these occurrences ranging between 0–100 m. Slope largely reflects the patterns observed in the regional model with a preference for locations with a slope component <20°, although here the majority of sites is found at 0–10°. In terms of distances from the coast we note that sites range from <100 m to > 5000 m from the modern coastline, with almost half of the sites (46.6%) located up to 500 m from the coast. Considering distance from freshwater sources, our analysis shows that 60% of the sites are found between 500–5000 m from a river source, with only 13.3% of the sites located within 500 m. It is known that tectonic and/or climatic events can alter river courses over millennia; however, here we have concentrated on major rivers with a well-established Quaternary evolutionary history. It is possible that the existence of smaller tributaries or perennial/seasonal streams that were not included in the analysis skews the actual distance from freshwater sources for these early sites. Finally, if we consider the maximum distance of these early sites from either the coastline or rivers and springs, we note that most of these archaeological localities are located within 5 to 10 km from either water source (Figs 4–6).

**Table 2:** Analysis outcomes of the main topographic patterns of Terminal Pleistocene and legacy Palaeolithic surface lithic assemblages from Cyprus.

**Elevation **	**No sites**	**%**
Elevation 0–100 m	7	46.6
Elevation 100–200 m	4	26.7
Elevation 200–350 m	3	20.0
Elevation >350 m	1	6.7
**Slope **	**No sites**	**%**
Slope 0–10 degrees	11	73.3
Slope 10–20 degrees	1	6.7
Slope >20 degrees	3	20.0
**Distance from coastlines **	**No sites**	**%**
Distance from coastline < 100 m	3	20.0
Distance from coastline 100–500 m	1	6.7
Distance from coastline 500–5000 m	7	46.6
Distance from coastline >5000 m	4	26.7
**Distance from rivers **	**No sites**	**%**
Distance from rivers < 100 m	0	0
Distance from rivers 100–500 m	2	13.3
Distance from rivers 500–5000 m	12	80.0
Distance from rivers >5000 m	1	6.7

Summary table of the analysis outcomes of the main topographic patterns of Terminal Pleistocene archaeological sites and locations of surface lithic assemblages of a potential Palaeolithic age on Cyprus.

We acknowledge that in these analyses we rely on the modern coastline and, presumably, archaeological localities may currently be under water as a result of post-glacial sea level changes. High-resolution bathymetric data are not readily available for the submerged landscape of Cyprus, limiting our understanding of the island’s underwater relief and pre-Holocene land extension. However, considering that our research timeframe is the last 125 ka, we should revisit the issue of presently submerged landscapes that were terrestrial landscapes during glacial periods. About 110 ka and then again 90 ka sea level had dropped as much as 50 m below present sea level. About 65 ka sea level had dropped as much as 90 m below present sea level, but the most dramatic drop is during the Last Glacial Maximum (about 22 ka) when sea level dropped as much as 120 m below present sea level. These presently submerged terrains consist of a shelf that can be fairly narrow or wide, depending on the shelf topography, the steeper the submerged coast topography, the more narrow the zone of interest. This zone is 1.5 km wide in the more precipitous areas and as much as 8 km wide in the more shallow shelfs. Most of the onshore analysed sites are found at a distance of around 10 km from the modern coast we assume that distance from the coast was not a controlling factor on location preferences for Pleistocene occupation of Cyprus. In their study of the Last Glacial Maximum in the Western Mediterranean, Barton et al. [53, 54] note that there is a general tendency for assemblages accumulating in base camps to predominate in a zone approximately 40–60 km from the contemporaneous coast, while ephemeral camps are located both seaward of the base camps, in a zone 0–15 km from the coast, and further inland, at 100–125 km from the coast. Thus, even though marine transgressions at the end of the Pleistocene may have altered distance of sites to the coast on Cyprus, some sites are expected to be located not directly associated with coastlines, but in the interior of the island.

Our analyses show that distance from a water source would have been an important parameter for site selection localities on behalf of Pleistocene hunter-gatherers. Indeed, on Cyprus, one can find the earliest securely dated water wells in the world (Early Holocene), which suggests that early populations on the island were aware of their availability and had good knowledge of how to best exploit the fresh water sources of the island [55]. Our knowledge of the spatio-temporal distribution of paleohydrological resources during episodes of climatic amelioration (increased/decreased rainfall) remains limited and for our analyses, we rely on modern day data. Despite these constraints and considering that permanent water sources require particular lithologies, hydrogeological environments and geomorphic settings [56–59], it is reasonable to expect that the distribution of freshwater sources in the past is likely broadly analogous with their modern distribution [60–64].

**Fig 7:**
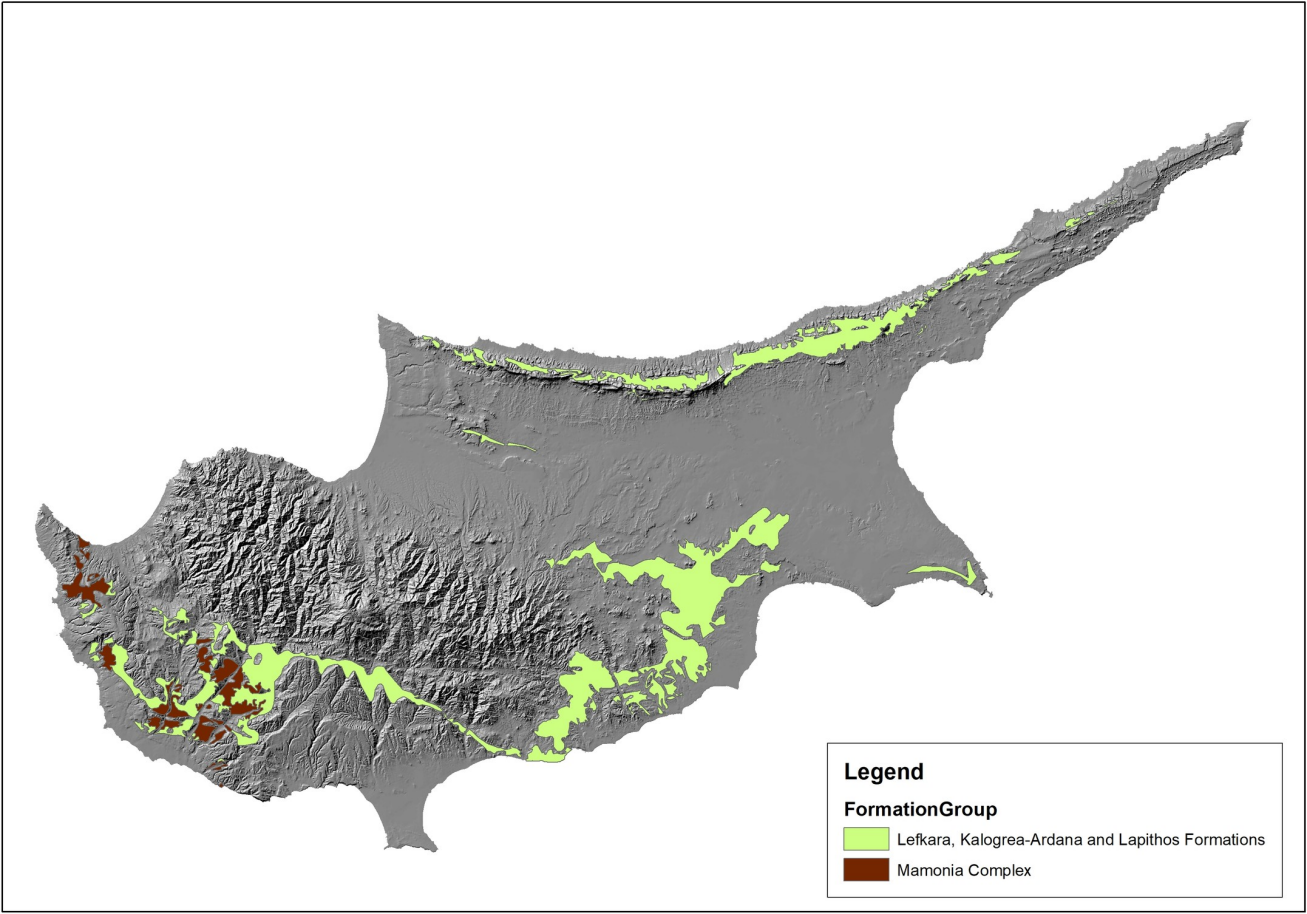
Chert-bearing geological formations of Cyprus. Original copyright with the authors.

Another key element in site location choices would have been lithic raw material availability. Lithic resources play an essential role in Palaeolithic lifeways as they provide the raw materials for stone tool production for the fulfillment of various subsistence-related everyday tasks. On Cyprus, chert is the main raw material resource with performance characteristics–‘knappability’, sharpness and durability–and fracture mechanics ideal for tool manufacture [65]. We included lithology as a parameter in our model, focusing particularly on chert-bearing geological formations (Fig 7). Most of the Cypriot sites used in the model appear to be located within these resource-rich zones, although it is not presently possible to estimate the exact distances to the various sources. Rarely, a site is found outside a chert-bearing formation; this may indicate that raw material acquisition could also take place at a distance, possibly from secondary sources. For example, Akrotiri *Aetokremnos* is located at a distance from any of the region’s chert-bearing geological formations but Kouris River, one of the major rivers of Cyprus, is located only a short distance from the site, suggesting hunter-gatherers could have procured material not necessarily from the rock outcrops but from rich river deposits, such as the active channel and flood plain deposits.

### Field survey results

**Fig 8:**
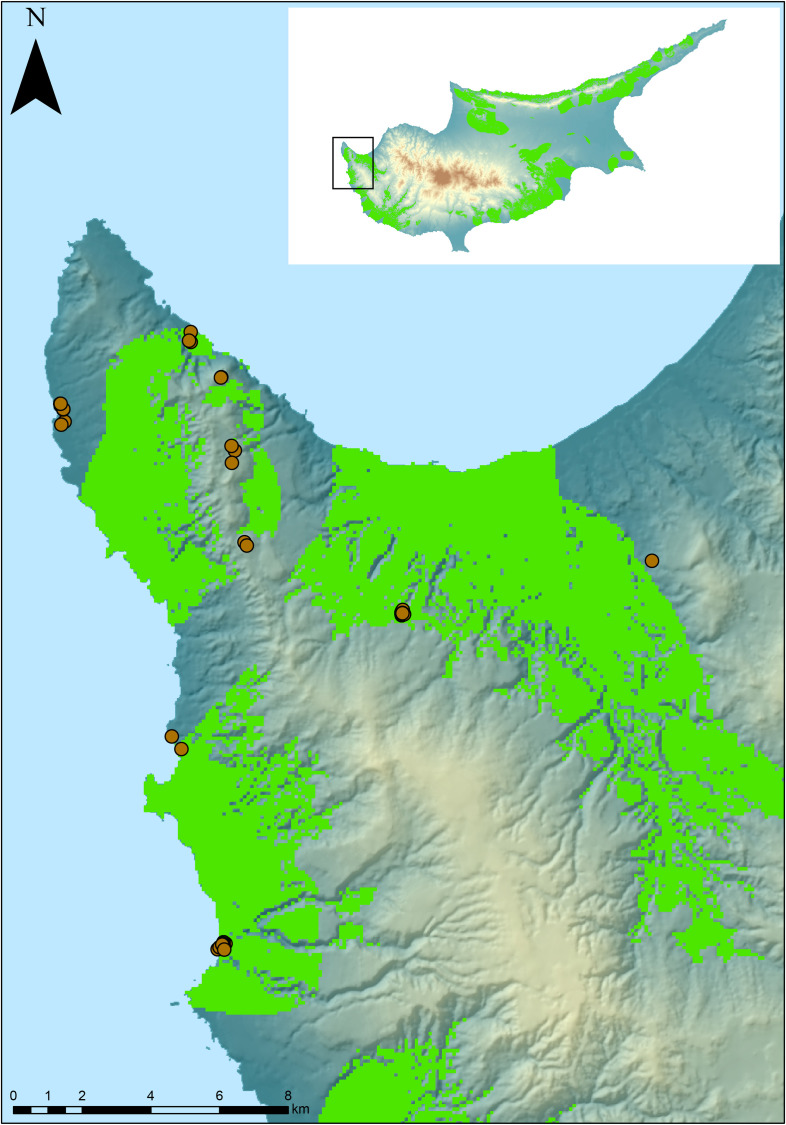
Archaeological localities identified during field surveys based on the predictive model outcomes. Archaeological localities identified during field surveys superimposed over predictive map (green area). Insert shows the location of surveyed region on the island of Cyprus. Original copyright with the authors.

A preliminary research-based field survey has been conducted as a spatially explicit island-scale assessment of the opportunities for Pleistocene human occupation of Cyprus. The survey used initial outcomes of the GIS model and targeted appropriate landscape features in an endeavor to collect field archaeological data that will feed back into the model. Although the GIS model considers the island as a whole, field surveys are only possible in the southern part of the island, which is currently under the effective control of the Republic of Cyprus. Over two seasons, key localities–as determined by the geospatial predictive model–across the island were located and investigated via intensive foot survey. Reconnaissance surveys targeted both the coastal zone and the interior of the island, as delineated by the GIS model, while also focusing on specific topographic features, such as rockshelters, rivers and Pleistocene-aged deposits. Surface lithic scatters as well as individual artefacts (isolated finds) were identified and recorded in various localities (Fig 8) confirming the expectations of the geospatial model. Based on a preliminary study of their morphological-typological characteristics, these chert stone tools likely range chronologically from at least the Epipalaeolithic to the Neolithic (Terminal Pleistocene–Early Holocene). The artefacts, mainly flakes and blades, are made from different types of chert. The majority of the identified archaeological localities have been found on a coastal setting, reflecting similar patterns to those observed from the Cypriot legacy collections and excavated Terminal Pleistocene sites.

Another important outcome of the field survey is the open-air character of the documented localities. Caves are not a common feature of the Cypriot landscape but several small rockshelters were noted during fieldwork. The team investigated those as they are assumed to preserve intact deposits. No evidence of prehistoric exploitation of those landscape features was recorded on any of the studied rockshelters. This outcome is an important negative confirmation of the predictive model highlighting its validity since most of the rockshelter sites are located on slopes >20% and are, hence, outside the model’s high probability zone. The identification of several lithic scatters of at least a Terminal Pleistocene age is significant as it adds to a modest corpus of data from Cyprus for this period. These sites begin to illustrate the true density of likely hunter-gatherer/forager sites on the island and show that mobile groups were exploiting and perhaps residing on Cyprus for several millennia, potentially earlier than the current archaeological record allows for.

## Discussion: Modelling the Pleistocene exploitation of Cyprus

Decision-making relies on the active engagement of humans with their environments and includes any choice among alternative courses of action that usually involve a set of feasible alternatives/options [66]. For Pleistocene hunter-gatherers exploiting a new territory, crucial decisions will involve subsistence strategies, resource acquisition and home base locale preferences at the very basic level, which in turn affect social interactions. Forward planning and advanced organisational capabilities enabled Pleistocene hunter-gatherers, for example, to successfully colonise northern latitudes during full interglacial conditions in the Middle Pleistocene [67]. Planning ahead provides a degree of cognitive plasticity that must not be underestimated. Whatever the difficulties of adapting to a new territory or environmental context, the fact that Pleistocene hunter-gatherers elsewhere were able to flourish in all climatic conditions and habitats argues for a high degree of resourcefulness [67–69].

The island of Cyprus has traditionally remained isolated from discussions concerning the pre-Holocene colonisation of islands. This is primarily a result of persisting narratives of the Mediterranean islands as marginal environments to a hunter-fisher-gatherer lifestyle. Archaeological discoveries of the last 15–20 years are slowly bringing to light evidence of the earliest phases of the Neolithic on the island. Unsurprisingly, however, this ‘marginality’ reasoning has discouraged archaeologists from actively searching for earlier, Pleistocene, sites on Cyprus. Using a geospatial predictive modeling approach, and keeping in mind the remarkable plasticity observed in hunter-gatherer societies (e.g. [70] and references therein), this paper proposes a systematic methodology for the study of the island’s Pleistocene exploitation. The geospatial model developed considered aspects of topography, such as elevation and slope, as well as environmental parameters, including fresh water and raw material sources. Although separately these parameters were not found to be of particular significance in site location, in combination they delineated areas, which were more attractive to Pleistocene exploitation. These areas are characterised by elevations <350 m and usually range between 0–100 m, slopes <20%, and mainly 0–10°, distance from the coast <500 m and freshwater sources 500–5000 m.

Proximity to water, especially freshwater, is important in the selection of site locations by hunter-gatherers on Cyprus and elsewhere. A recent study from Australia [59] demonstrated the importance of water in Palaeolithic societies by showing that 84% of archaeological sites >30 ka old are within 20 km of modern permanent water. In the Arabian subcontinent, approximately 45% of Lower and Middle Palaeolithic sites are located within 10 km of drainages and 68% and 58% respectively within 20 km of drainages [71]. A progressive drop in the number of recorded archaeological sites occurs as distance from major drainage features increases [72]. Cyprus today experiences a semi-arid/arid climate but several major permanent rivers transcend its territory. Numerous springs further contribute to fresh water accessibility. Although the island’s Pleistocene hydrological network is not well understood, the distribution of freshwater sources in the past is likely broadly comparable with their modern distribution. Permanent water points, such as rivers and springs, require particular lithologies, hydrogeological environments and geomorphic settings [56, 57]. Thus, while it is possible that smaller water sources (e.g. small springs, rockholes) may activate or deactivate over time, their general location as well as the location of larger water points can be safely assumed to be relatively constant. Apart from being vital to human survival, freshwater sources are important landscape features for human subsistence as they are rich with aquatic plants and prey species such as fish, shellfish and turtles and they also attract faunal species, thus, contributing to energy intake as locales for finding hunting prey and carcass scavenging [73–75].

The coast would have also contributed to the subsistence supply for Pleistocene hunter-gatherers. Our model showed that distance to the coast functioned as an important parameter in site location preferences. This outcome adds to an existing body of evidence regarding the exploitation of the Cypriot coastline by Epipalaeolithic groups as attested in sites such as Akrotiri *Aetokremnos* [33, 76], Nissi Beach and Akamas *Aspros* [35] on southern Cyprus. The faunal assemblage from *Akrotiri Aetokremnos*, the earliest known Terminal Pleistocene site on Cyprus (12 ka), provides evidence for the consumption of shellfish and small quantities of fish, alongside pygmy hippos and elephants [33, 76].

The distribution, abundance and quality of raw material sources for stone tool manufacture conditions how these resources can be exploited [77–80]. An examination of the geology of Cyprus shows an abundance of chert sources across the island [81, 82]; the Lapithos chalks with cherts border the northern Kyrenian range, while the larger Lefkara formation lies within the sedimentary succession that encircles the Troodos Mountains (see also [83] for a detailed description of the formations). Surveys in central and western Cyprus have identified over fifty chert and jasper sources, ranging in quality, located along the pillow lava and sedimentary boundary of the Troodos foothills [36, 37, 84]. Raw material procurement while undertaking subsistence activities minimises direct costs [85] and the abundance of sources in these localities would have facilitated an embedded strategy in these areas, allowing Pleistocene humans to carry out other activities in the knowledge that a chert source was in the vicinity whenever new tools were required. Our model shows that although *immediate* access to a good quality source of stone tool raw material was not a determining factor in site location, distance from such a resource influenced site preferences. All documented archaeological localities are found within geological zones with chert outcrops.

While field investigations have confirmed the power of our predictive analyses to successfully identify areas that preserve cultural material of a Pleistocene age, it has also highlighted zones of low probability to unearth prehistoric archaeology. High elevations, over 350–500 m, can be excluded from investigations targeting a pre-Holocene occupation of Cyprus. The availability of Pleistocene fauna that would have been available for hunting, namely pygmy hippos and elephants, at low elevations, supports this predictive outcome. We discussed elsewhere in this paper the plasticity of the human species to successfully adapt to resource availability at a range of environmental circumstances. By excluding certain areas from future investigations, our model does not deny Pleistocene hunter-gatherers on Cyprus the ability to occupy and explore the full extent of the island. Rather, it suggests localities that would have been preferentially selected for repeated visits and are, thus, more likely to preserve material evidence of prehistoric choices.

Movement is a primary means for humans to read and experience the landscape and connect with one another [24, 86, 87]. The manifestation of evidence of movement on the landscape largely depends on the frequency and duration of the routes used, possible intentionality of making them visible, and, their chance conservation through time [87]. The selection of a location for resource exploitation or settlement is a reflection of decisions that are influenced by the affordances of the natural environment [88, 89] but also by personal preference and social factors [90]. In the case of Pleistocene Cyprus, we are called to make sense of hunter-gatherer adaptation and human-insular environment relationship by looking for low density, dispersed lithic scatters that are notoriously difficult to detect in the field.

## Conclusions

GIS-based analyses were performed on Pleistocene Cyprus as a heuristic model applied to determine likely hotspots of early human activity on the island. Topography alone fails to provide sufficient predictive markers; a variety of factors, primarily freshwater sources, lithic raw material availability and the distribution of floral and faunal resources, as well as social interactions, all played key and dynamically varying roles in these processes throughout the Pleistocene. The results of the analyses can be used predictively to identify areas worthy of investigation for their potential to yield previously unrecognised archaeological sites and delimit the pre-Holocene colonisation of the eastern Mediterranean island. Preliminary field surveys confirm the validity of the model and provide new data concerning early human presence on the island. We view the model we have presented here as a useful first step in attempting to approach the question of when and how early humans reached Cyprus and contribute to discussions of the potential characteristics of hominin lifeways during the Pleistocene (see also [7]). Further work and, especially, high-resolution palaeoenvironmental and palaeoclimatic records are required to refine these initial outcomes and excavations to ground truth the predictions. We are hopeful that our study will help set the foundations for targeted and multi-disciplinary Pleistocene research in Cyprus and, alongside other recent advances in archaeological thought, contribute to the island colonisation thematic in a part of the world, where this topic remains, peculiarly, largely unexplored.

## Supporting information

S1 Table Summary table of archaeological sites of a Pleistocene age documented in the Eastern Mediterranean. (XLSX)
